# Adults with Phenylketonuria have suboptimal bone mineral density apart from vitamin D and calcium sufficiency

**DOI:** 10.3389/fendo.2025.1488215

**Published:** 2025-02-14

**Authors:** Beatrice Hanusch, Anne Schlegtendal, Corinna Grasemann, Thomas Lücke, Kathrin Sinningen

**Affiliations:** ^1^ Research Department of Child Nutrition, University Children’s Hospital of Ruhr-University Bochum, St. Josef-Hospital, Ruhr-University Bochum, Bochum, Germany; ^2^ Department of Paediatric Pulmonology, University Children’s Hospital of Ruhr-University Bochum, St. Josef-Hospital, Ruhr-University Bochum, Bochum, Germany; ^3^ Department of Rare Diseases, University Children’s Hospital of Ruhr-University Bochum, St. Josef-Hospital, Ruhr-University Bochum, Bochum, Germany; ^4^ Department of Neuropediatrics and Metabolism, University Children’s Hospital of Ruhr-University Bochum, St. Josef-Hospital, Ruhr-University Bochum, Bochum, Germany

**Keywords:** aromatic amino-acids, bone turnover, dietary compliance, calciotropic hormones, oxidative stress, dietary habits

## Abstract

**Introduction:**

Improvement of early diagnosis and treatment in patients with Phenylketonuria (PKU) allowed for healthy survival into adulthood of these patients, but non-neurological health impairments of unknown etiology emerged. One of these is impaired bone health that manifests in adolescence and adulthood, potentially depending not only on treatment adherence but also on additional lifestyle factors and nutrition.

**Methods:**

Eighteen adults with PKU (18.3–51.6 years, ♀ n = 11) and 19 age- and gender-matched controls (18.3–54.9 years, ♀ n = 10) participated in the study. Bone metabolism–related parameters (BMRPs) in plasma, serum, and urine were analyzed. Dietary habits and lifestyle factors were obtained from questionnaires; a 6-min walk test and a dual X-ray absorptiometry measurement at two sites were performed. Phenylalanine (Phe) serum concentrations from the 5 years prior to study participation were collected from the patients’ charts.

**Results:**

Patients had reduced bone mineral density (BMD) T-score in hips (−0.67 ± 1.05) and lumbar spine (−0.71 ± 1.11, both p = 0.018). Most BMRPs in plasma, serum, and urine, as well as markers of oxidative stress did not differ from healthy controls. Whereas 89% of adults with PKU were vitamin D–sufficient, only 68% of controls reached vitamin D sufficiency. 25-Hydroxy vitamin D concentration was significantly higher in adults with PKU than that in controls [33.1 ng/mL (26.2–40.3) vs. 23.4 ng/mL (17.2–24.9); p < 0.001], whereas parathyroid hormone concentrations showed no difference [PKU: 27.6 pg/mL (19.6–42.8) vs. Co: 36.1 pg/mL (29.2–40.8); p = 0.089]. Neither Phe blood concentration nor dietary habits or lifestyle factors were associated with BMD in regression analysis.

**Conclusion:**

Neither dietary habits nor lifestyle factors showed an association with BMD in this group of adults with PKU, whereas BMD was reduced.

## 1 Introduction

Phenylketonuria (PKU; OMIM (Online Mendelian Inheritance in Man) #261600) is an inborn error of the phenylalanine hydroxylase with an incidence of 19.3/100,000 newborns in 2020 in Germany (1, 2). The reduced activity of the enzyme results in the accumulation of Phenylalanine (Phe) and its metabolites in blood and brain, leading to irreversible intellectual disability, motor, and developmental deficits, if left untreated (1). Diagnosis via newborn screenings and subsequential early start of treatment with reduction of Phe intake from natural protein and supplementation of amino acids (AA) can prevent the development of most symptoms (1, 3). Additionally, tetrahydrobioterin (BH_4_), the co-factor of phenylalanine hydroxylase, is available as therapeutic agent for patients if they respond to the treatment. This allows them to either increase their intake of natural protein by ≥ 100% and/or achieve better metabolic control (1). A strict dietary regime is important during maturation of the brain; thereafter, higher blood Phe concentrations can be tolerated (1). In children and adolescents with PKU, quality of life is significantly impaired by their reduced enjoyment of food, in part due to AA supplements (4). As brain development progresses in adolescents, diet restrictions are relaxed and higher Phe concentrations are tolerable (1). Due to the impairment of quality of life observed in adolescents, a less strict diet is usually followed in adolescents and adults with PKU. To reduce the intake of natural proteins, adults tend to adopt a vegan-like diet, with optional vitamin- and mineral-enriched AA supplement consumption, depending on activity level of the phenylalanine hydroxylase (5). The leading goal in therapy of patients with PKU is the prevention of neurocognitive deficits (1); however, other health impairments of unknown etiology occur (6). One of these health impairments is reduced bone mineral density (BMD) of patients with PKU (6). In pediatric patients with PKU, a low BMD Z-score was observed in 28%–45% of patients, even without family history of osteoporosis (7, 8). In adults with PKU, BMD Z-score below −2 in lumbar spine, femoral neck, total proximal femur, or total body was observed in 1.6%–5.5% of patients, whereas overall mean BMD Z-scores were significantly lower compared to reference, without higher proportion of fractures (9). Factors for impaired bone health could be dietary compliance as high blood Phe is associated with reduced bone quality and higher spontaneous osteoclastogenesis (10, 11), whereas the intake of AA supplements might affect bone mineralization negatively due to high renal acid load (12–16). Energy and protein intake play a crucial role in bone formation. In fact, 21% of total body bone area could be explained by protein intake in children and adolescents (17–19). However, the impact of protein intake on bone formation is lower in adults (18). Additionally, physical activity influences not only bone mass accrual but also maintenance even in patients with low BMD (17, 19). Furthermore, various influencing factors on BMD have been studied in the general population throughout the last years (17, 19). One of these is educational attainment which is associated with higher BMD, although this may be mediated partially by heightened coffee intake and reduced consumption of processed meats (20). Because bone can be influenced by various factors, we analyzed involvement of lifestyle factors and dietary compliance in adult patients with PKU and healthy controls.

## 2 Materials and methods

### 2.1 Subjects

Patients were recruited during routine check-up at the outpatient clinic in the Children’s Hospital in Bochum. As 32%–77% of adults are lost to follow-up of care in specific centers for PKU care (21), additional recruitment by word of mouth, advertisement in prints for interest groups, and posts on social media were used to reach patients outside of specific clinical care centers. Patients were asked to bring a friend, partner, or sibling as control. Missing controls were recruited by word of mouth. Nineteen age- and sex-matched controls (**Table 1**) were included into the study on the same day as the patients with PKU.

**Table 1:** Characteristics of the participating patients with Phenylketonuria (PKU) and controls (Co).

Parameter	PKU	Co	p	Effect size
n	18	19	–	–
Age (years)	37.9 (23.6–45.0)	38.3 (23.8–46.5)	0.869	−0.03^⊖^
Female, n (%)	11 (61%)	10 (53%)	0.603	–
**Height (cm)**	**167.3 ± 9.3**	**176.4 ± 9.7**	**0.006**	**−0.95^~^**
Weight (kg)	70.1 ± 17.3	76.4 ± 14.5	0.235	−0.40**^~^**
BMI (kg/m²)	23.6 (22.1–26.8)	24.5 (21.4–26.3)	0.916	−0.02^⊖^
Smoking habits, n (%)				
Never	10 (56%)	11 (58%)	0.708^a^	–
Not any more	2 (11%)	4 (21%)		
Sometimes	3 (17%)	1 (5%)		
Daily	3 (17%)	3 (16%)		
Heavy smoking according to RKI [26]	2 (11%)	2 (11%)	1.000^a^	–
Pack-years	0.0 (0.0–2.5)	0.0 (0.0–10.0)	0.711	−0.06^⊖^
Not fit 6MWT, n (%)	6 (33%)	3 (16%)	0.269^a^	–
ΔDistance 6MWT	16.3 (−48.0–70.7)	50.3 (3.0–129.0)	0.134	−0.25 ^⊖^
**SES**	**6.7 ± 2.2**	**8.6 ± 2.6**	**0.021**	**−0.79^~^**
WISH	60.6 ± 16.4	59.4 ± 18.3	0.846	0.07**^~^**
**Protein-rich foods (%^#^)**	**8.6 (3.5–15.0)**	**32.3 (21.3–48.8)**	**< 0.001**	**−0.58** ^⊖^
Women who were pregnant before, n (%)	3 (27%)	5 (50%)	0.387^a^	–
Phe during last 5 years (µmol/L)	591 ± 340	–	–	–
Patients with mean blood Phe > 600 µmol/L, n (%)	7 (47%)	–	–	–

a Fisher’s exact test; ^~^Cohen’s d; ^⊖^Pearsons r; ^#^ratio of protein- to carbohydrate-rich food items. 6MWT, 6-min walk test; ΔDistance 6MWT, difference between walked distance and predicted distance 6MWT (m); BMI, body mass index; RKI, Robert Koch Institute; SES, socioeconomic status; WISH, World Index for Sustainability and Health; Phe, Phenylalanine. Data are reported as median (25th–75th percentile) (non-normal distribution) or as mean ± standard deviation (normal distribution). Significant results are marked bold.

Classical PKU was defined as either being treated with AA supplements and/or BH4 supplementation or untreated Phe levels > 600 μmol/L. Patients with hyperphenylalaninemia do not require treatment to maintain blood Phe levels < 600 μmol/L (1). Patients with PKU and hyperphenylalaninemia were invited to participate, as all patients did either exceed 600 µmol/L of Phe or were treated with AA supplements, we can classify all included patients with PKU as patients with classical PKU.

A written informed consent was given by all participants. The study was approved by the Ethics Committee of the Ruhr-University Bochum (No. 20-7008). The number of eligible data of the parameters varied due to missing or small sample volumes in patients and controls.

### 2.2 Anthropometric data, lifestyle, and nutrition

Dietary habits were recorded by a validated self-administered food frequency questionnaire on 53 food and drink items previously used in the German Health Interview and Examination Survey for Adults 1 (DEGS1) (22, 23) and extended by current use of AA supplementation in patients with PKU. Dietary quality was calculated by the World Index for Sustainability and Health (WISH) using the calculated daily intake of the 53 items of the food frequency questionnaire (24, 25). For further analysis of food intake, healthy and unhealthy subsections were calculated. Healthy subsection included vegetables, fruits, nuts, fish, poultry, whole grains, fish, milk and dairy products, and eggs, whereas unhealthy subsection included red meat, saturated fats, sugar sweetened beverages, sugary and salty snacks, and spreads (24). As mentioned in the introduction, adult patients with PKU tend toward a vegan diet, excluding most protein-rich foods and substitute these with carbohydrate-rich foods, which, in turn, might be disadvantageous for bone health. Therefore, food items were categorized into protein-rich (milk and dairy products, eggs, white and red meats, fish, and legumes) and carbohydrate-rich (fruit juices, fruits, vegetables, grains, potatoes, rice, sweets, and snacks) items, and proportions of these were calculated. As fats were only estimated from butter and oil usage, proportions of these were not calculated.

Dietary compliance of patients with PKU was recorded from blood Phe concentrations during the last 5 years prior to study participation. As all participants were adults at time of study participation, the target Phe levels during the 5 years before recruitment were 120–600 µmol/L, according to the European guidelines (1). For evaluation, the mean blood Phe concentration during the last 5 years was calculated, and patients were grouped into good compliance when their mean blood Phe concentration during the 5 years stayed below 600 µmol/L or poorly compliant, when their mean blood Phe concentration during the 5 years was above 600 µmol/L.

Lifestyle factors such as highest level of educational, socio-economic factors, smoking habits, current medication, and physical activity were examined by self-administered questionnaire previously used in DEGS1 (23). Fractures during the last 12 months prior to study participation were recorded by self-administered questionnaire and examination by the responsible physician. In female participants, the number of pregnancies and births, the use of hormonal contraceptives, the status of menopause (defined as no menstruation in the prior 12 months), and hysterectomy or ovariectomy were recorded. Smoking habits were recorded as previous and current smoking, with additional classification of heavy smokers as defined by Robert Koch Institute (RKI) (26), and pack-years were calculated (27). Socioeconomic status (SES) was calculated on the basis of the work by Lampert et al. (2013) (28), including highest education and employment status of the main earner among the household members. Here, household income was not included in the SES; therefore, the lowest SES possible was 2 points, with the highest points being 14. A 6-min walk test (6MWT) was used for further examination of physical fitness. Participants were instructed to walk along a corridor of 36 m for 6 min as quickly as possible, whereas the distance was recorded by study personnel. Participants were defined as physically active if they achieved > 96% of the predicted distance in the 6MWT calculated by considering age and gender of participants (29, 30).

BMD from dual X-ray absorptiometry (DXA) was either acquired from records of patients with PKU or measured wherever indicated, expressed as T- and in Z-scores at the left proximal femur (hip) and at the lumbar spine (L1-L4; LS). DXA device used was Hologic Delphi C (Hologic Medicor GmbH, Berlin, Germany), each standardized for gender, weight, and ethnicity by the provider.

### 2.3 Sampling and biochemical analyses

Unfasted venous blood was drawn in ethylenediaminetetraacetic acid (EDTA) monovettes and monovettes for serum collection (Kabe, Nümbrecht-Elsenroth, Germany). EDTA blood was centrifuged at 3,000 rpm at 4°C for 10 min. Serum was incubated at least 30 min at room temperature to clot, centrifuged at 3,000 rpm at 4°C for 10 min, and the plasma and serum were stored at −80°C until analysis. Spot urine samples were collected and stored at −80°C until further analysis. In plasma, serum, and urine bone metabolism–related parameters (BMRPs), markers of oxidative stress and anti-oxidative vitamins were analyzed. Intact parathyroid hormone (PTH), 25-hydroxy vitamin D3 (25-OH D), cross-linked C-telopeptide of type I collagen (CTX), deoxypyridinoline (DPD), pyridinoline (Pyr), insulin-like growth factor 1 (IGF1), total alkaline phosphatase (ALP), and osteocalcin (OCN), as well as anorganic phosphate, calcium, and creatinine in urine and serum and were analyzed at the MVZ Dr. Eberhard & Partner GbR Dortmund, Germany.

Analytical assays for anorganic phosphate in serum and urine, calcium in serum and urine, ALP, creatinine in serum and urine, and C-reactive protein (CRP) were performed on an automated clinical chemistry module cobas c701 (Roche Diagnostics GmbH, Mannheim, Germany). 25-OH D, IGF1, CTX, and PTH were analyzed via ECLIA technology on an automated cobas 8000 e801 analytical unit (Roche Diagnostics, Mannheim, Germany). OCN was analyzed on the fully automated immunodiagnostic Liaison XL platform (DiaSorin, Saluggia, Italy) using CLIA technology. Here, OCN assay detects both the intact OCN (1–49) and the N-terminal midfragment (1–43). Intra-assay and inter-assay coefficients of variation (CVs) were, respectively, the following: 0.6% and 0.7% for phosphate in serum, 0.6% and 1.1% for calcium in serum, 0.7% and 0.8% for creatinine in serum, 0.4% and 1.1% for ALP, 2.6% and 3.5% for 25-OH D, 1.2% and 1.5% for CTX, 0.8% and 1.4% for IGF-1, 0.9% and 1.0% for PTH, 1.4% and 2.0% for OCN, 0.6% and 0.8% for CRP, 1.2% and 1.9% for urinary calcium, 0.9% and 2.4% for urinary creatinine, and 0.9% and 1.2% for urinary anorganic phosphate. Analysis of Pyr and DPD was performed on a modular Ultra high pressure liquid chromatography (UHPLC) system with fluorescence detector (Waters Acquity H-Class system, equipped with binary solvent manager, autosampler with flow through needle and column manager, all from Waters GmbH, Eschborn, Germany) using an Acquity UPLC HSS T3 Column (1.8 µm, 2.1 × 100 mm, Waters GmbH, Eschborn, Germany). Separation was achieved using a gradient of mobile phase A (0.2% heptafluorobutyric acid in water) and mobile phase B (acetonitrile) at a flow rate of 0.5 mL/min. Excitation wavelength was 290 nm, and emission wavelength was 395 nm. After hydrolysis of 250 µL of the sample material with 250 µL of 25% hydrochloric acid and 50 µL internal standard (#48004) at 100°C for at least 12 h, extraction was achieved by addition of 2.5 mL of extraction buffer (#48005, both, Chromsystems Instruments & Chemicals GmbH, Gräfelfing/Munich, Germany). The sample clean-up columns (#48008) were primarily conditioned with 2.0 mL of wash buffer (#68007). Subsequently, SPE-Columns were loaded with the hydrolyzed sample material and washed three times with 2.5 mL of wash buffer. Elution of the analytes was achieved using 1 mL of elution buffer (#68010, all Chromsystems Instruments & Chemicals GmbH, Gräfelfing/Munich, Germany). The eluate (5 µL) was injected into the HPLC system. The calibration standard (#48003) and the two control levels (#0046 and #0047, all Chromsystems Instruments & Chemicals GmbH, Gräfelfing/Munich, Germany) were prepared analogous to the patient sample material for daily calibration and quality control. Intra-assay and inter-assay CVs were, respectively, 1.1% and 2.9% for low levels (315 µg/L) and 1.5% and 3.3% for high levels (911 µg/L) of Pyr, as well as 1.6% and 3.5% for low levels (63 µg/L) and 1.5% and 3.2% for high levels (167 µg/L) of DPD. Urinary analytes calcium, Pyr, and DPD were normalized to creatinine excretion. In serum, osteoprotegerin (OPG) and tartrate-resistant acid phosphatase 5b (TRAP) were measured by using commercially available ELISA kits (OPG: Abcam Limited, Cambridge, UK; TRAP: BoneTRAP^©^ Immunodiagnostic Systems, Boldon Colliery, UK) according to the manufacturer’s specifications.

25-OH D was used for evaluation of vitamin D sufficiency, as suggested by the Institute of Medicine (IOM) (31). To evaluate bone health, bone pathology harbinger (BPH) score was calculated as suggested by Schündeln et al. (32). For this score, one point was given if the levels of PTH, OCN, ALP, or DPD were outside the normal range, knee pain on exercise or spontaneous back pain occurred, and if BMD Z-score in any area was below −2. The BPH was calculated if at least five of these items were available, and points were summed up and divided by available items to achieve a score between 0 and 1.

Total oxidative status was measured as total lipid peroxides using a commercially available photometric test system (Immuchrom GmbH, Heppenheim, Germany) according to the manufacturer’s instructions. Intra-assay and inter-assay CVs were 3.6% each for total lipid peroxides. Additionally, vitamin E (Vit E) and vitamin C (Vit C) were measured. Analysis of Vit E was performed on a modular UHPLC system with dual wavelength UV-VIS detector (LC-20ADXR pump, DGU-20A3R degassing unit, SIL-20ACXR autosampler, CTO-20AC column oven, SPD-20AV UV-VIS detector, and CBM-20A communications bus module, all from Shimadzu Deutschland GmbH, Duisburg, Germany) using the Conformité Européenne - In vitro diagnostics (CE-IVD) validated Chromsystems One Step Vitamin A and E in Serum/Plasma – UHPLC analysis kit (#34900/UHPLC, Chromsystems Instruments & Chemicals GmbH, Gräfelfing/Munich, Germany). Mobile phase and UHPLC column are part of the analysis kit. UV detection was performed at 295 nm. Serum (100 µL) was vortexed with 200 µL of the pre-mixed solution (#34966, Chromsystems Instruments & Chemicals GmbH, Gräfelfing/Munich, Germany) for 30 s and centrifuged for 10 min at 14,000 U/min. The supernatant (5 µL) was injected into the HPLC system. Intra-assay and inter-assay CVs were, respectively, 2.3% and 4.6% for low levels (9 mg/L) of Vit E and 1.0% and 4.7% for high levels (19 mg/L) of Vit E. Analysis of Vit C was performed on a Waters Alliance e2695 HPLC system with dual wavelength Waters 2489 UV-VIS detector (Waters GmbH, Eschborn, Germany) using the CE-IVD–validated Chromsystems Vitamin C in Plasma/Serum – HPLC analysis kit (#65065, Chromsystems Instruments & Chemicals GmbH, Gräfelfing/Munich, Germany). Mobile phase and HPLC column are part of the analysis kit. UV detection was performed at 245 nm. For sample preparation, 100 µL of serum and 100 µL of Internal Standard (#65044, Chromsystems Instruments & Chemicals GmbH, Gräfelfing/Munich, Germany) were vortexed for 30 s and centrifuged for 5 min at 14,000 U/min. The supernatant (20 µL) was injected into the HPLC system. The kit calibration standard (#65003) and the two control levels (#0074, both Chromsystems Instruments & Chemicals GmbH, Gräfelfing/Munich, Germany) were prepared analogous to the patient sample material for daily calibration and quality control. Intra-assay and inter-assay CVs were, respectively, 2.1% and 3.5% for low levels (6 mg/L) and 3.2% and 2.5% for high levels (21 mg/L) of Vit C.

### 2.4 Statistical analyses

The statistical software package IBM^®^ SPSS^®^ Statistics for Windows, version 29.0 (IBM Corp., Armonk, NY, USA), was used for the statistical analyses. Descriptive data were analyzed by the Chi-squared test or, where applicable, by Fisher’s exact test. Shapiro–Wilk test was used to evaluate for normal distribution in small groups. The QQ plots were used to test for normal distribution in groups larger than n = 10. Normally distributed data were analyzed using parametric tests. Between-group comparisons of normally distributed data were analyzed with Student’s t-test; for comparison to general population, one sample t-test was used (9, 33). For normally distributed data, effect size was calculated using Cohen’s d for groups larger than 20 and Hedges g for groups smaller than 20 observations. Non-normally distributed data were analyzed using non-parametric tests (Mann–Whitney test). Non-normally distributed data effect sizes were calculated by Pearson’s r. Values of p < 0.05 were considered significant. Linear regression analysis with heteroscedasticity consistent co-variance matrix (HC3) was applied to evaluate associations between potential influencing factors and BMD. Normally distributed data are presented as mean ± standard deviation (SD), non-normally distributed data as median (25th–75th interquartile range). Results from DXA scans, height, and weight were compared to population by one-sample t-test.

## 3 Results

### 3.1 Study population characteristics

Eighteen adult patients with PKU and 19 age- and gender-matched controls participated in the study (PKU: 18.3–51.6 years; Co: 18.3–54.9 years; **Table 1**). Neither smoking habits nor body mass index (BMI), current education, or fitness according to 6MWT differed between healthy controls and patients with PKU (**Table 1**). Although, patients with PKU were significantly shorter than controls (p = 0.006 for both sexes, **Table 1**; female PKU: 161.9 ± 6.8 cm vs. Co: 169.5 ± 5.5 cm, p = 0.012, Hedges’ g = −1.17; male PKU: 175.7 ± 5.6 cm vs. Co: 184.0 ± 7.4 cm, p = 0.028, Hedges’ g = −1.17), patients with PKU were of normal height for the general population and weight did not differ between groups [**Table 1**, **Supplementary Table S1** (33)]. Controls had a significantly higher SES than patients with PKU (p = 0.021, **Table 1**). One control subject reported a bone fracture within the 12 months prior to participating in the study; none of the patients with PKU reported any fractures during the past 12 months. Nutrition, as evaluated by WISH, did not differ between patients with PKU and controls, whereas patients with PKU consumed significantly smaller proportions of protein-rich foods and significantly higher proportions of carbohydrate-rich foods, compared to controls. While four control subjects were menopausal, no patient was (data not shown).

Blood Phe concentrations during the 5 years prior to study participation were available from 15 patients with PKU who had a mean blood Phe concentration of 591 µmol/L (± 340 µmol/L; min. 170 µmol/L; max. 1,500 µmol/L) during this time period (**Table 1**).

### 3.2 Bone metabolism–related parameters and parameters of oxidative stress in patients with PKU and controls

**Figure 1:**
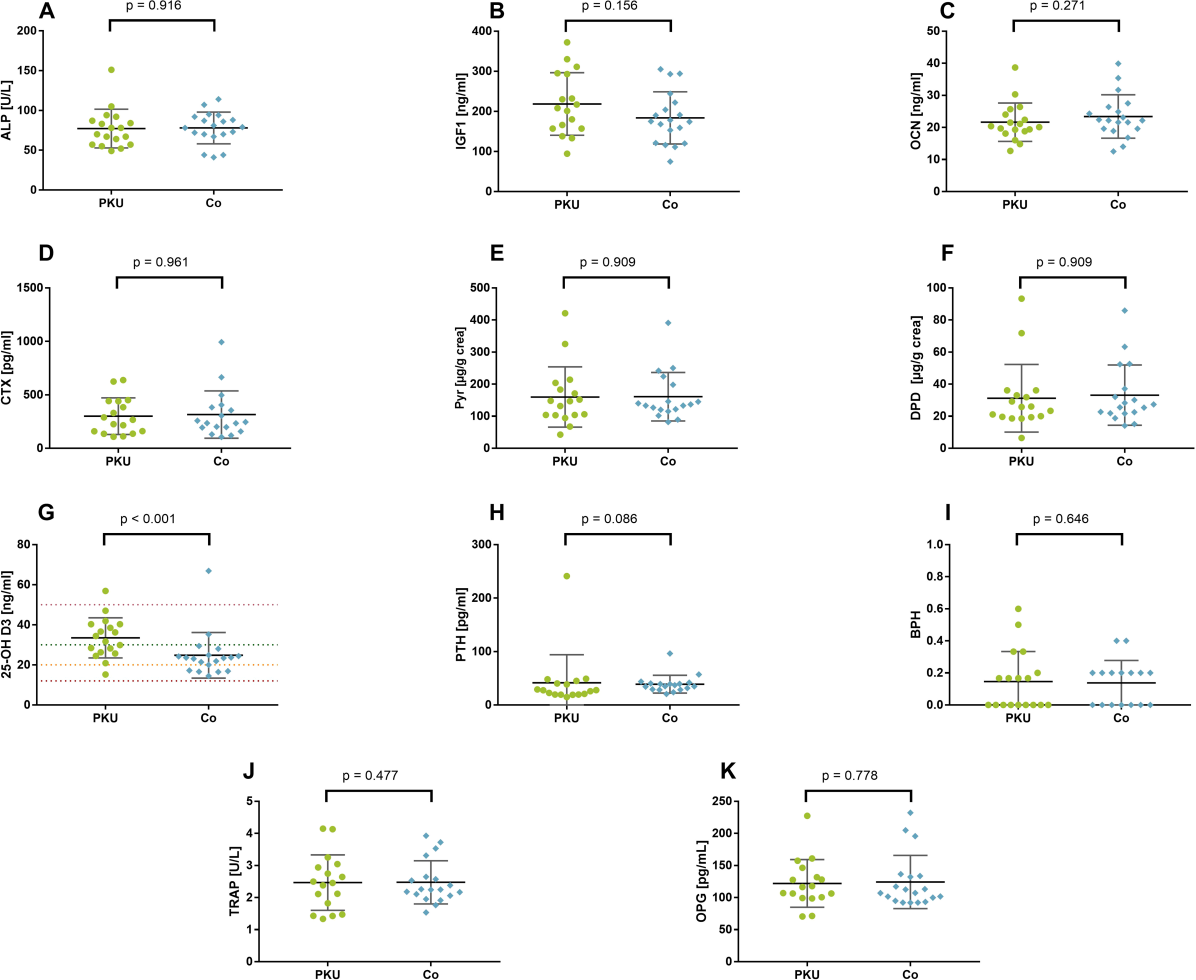
Bone metabolism–related parameters in patients with phenylketonuria (PKU) and controls (Co). Bone formation marker **(A–C)**: total alkaline phosphatase [ALP; **(A)**], insulin-like growth factor 1 [IGF1; **(B)**], and osteocalcin [OCN; **(C)**]. Bone resorption markers **(D–F)**: cross-linked C-telopeptide of type I collagen [CTX; **(D)**], pyridinoline [Pyr; **(E)**], deoxypyridinoline [DPD; **(F)**]. Calciotropic hormones **(G–I)**: 25-hydroxy vitamin D3 [25-OH **(D, G)**] with lines depicting deficiency (red), insufficiency (orange), sufficiency (green), and upper margin for sufficiency (pink); parathyroid hormone [PTH; **(H)**]; bone pathology harbinger [BPH; **(I)**]; osteoprotegerin [OPG; **(J)**]; tartrate-resistant acid phosphatase 5b [TRAP; **(K)**].

Neither the bone formation markers ALP, IGF1, and OCN nor the bone resorption markers Pyr, DPD, and CTX or BPH differed between the groups of PKU and Co (**Figure 1**). Only 25-OH D was significantly higher in patients with PKU [33.1 ng/mL (26.2–40.3)] than in controls [23.4 ng/mL (17.2–24.9); p < 0.001; r = −0.53], without a significant difference between the groups in PTH plasma concentration [PKU: 27.6 pg/mL (19.6–42.8) vs. Co: 36.16 pg/mL (29.2–40.8); p = 0.089; r = −0.29]. Although, no significant difference in vitamin D sufficiency can be reported (p = 0.114), 89% of the participants with PKU were vitamin D–sufficient, whereas 68% of controls were sufficient in vitamin D (data not shown). One participant in each group had 25-OH D serum concentration above 50 ng/mL but below 100 ng/mL. Parameters of oxidative stress, Vit C, and Vit E did not differ between patients with PKU and controls (**Supplementary Table S2**).

BMRP did not differ between patients with mean Phe below 600 µmol/L during the 5 years prior to study participation (good compliance) and those with mean Phe above 600 µmol/L or no measurements of Phe (poor compliance, **Supplementary Table S3**). Only Vit C was higher in patients with good compliance (12.3 ± 2.7 mg/L) compared to those with poor compliance (8.7 ± 3.4 mg/L, p = 0.031, Hedges’ g = 1.10), whereas CRP tended to be lower in patients with good compliance [0.06 mg/dL (0.04–0.17)] compared to those with poor compliance [0.21 mg/dL (0.09–0.45), p = 0.083, r = −0.41].

### 3.3 Bone mineral density in patients with PKU

DXA measurements were only available for patients with PKU. Out of the 18 patients, 17 had calculated T-scores for hips and LS, whereas Z-scores were available for all 18 patients. T-scores and Z-scores are depicted in **Table 2**. T-scores were significantly lower than zero, whereas Z-scores were only marginally reduced.

**Table 2:** Results from dual X-ray absorptiometry scans of patients with Phenylketonuria (PKU) in hips and lumbar spine (LS) compared to healthy 30-year-old reference population (T-score).

		PKU	p	Effect size
Hip	**T-score**	**−0.668 ± 1.05**	**0.018**	**−0.61^†^**
	Z-score	−0.419 ± 1.13	0.133	−0.36^†^
LS	**T-score**	**−0.712 ± 1.11**	**0.018**	**−0.61^†^**
	Z-score	−0.517 ± 1.16	0.075	−0.43^†^

^†^Hedges’ g. Data are reported mean ± standard deviation (normal distribution). Significant results are marked bold.

Eight patients (47.1%) with PKU had a T-score in the hip below the margin of −1 standard deviations and, therefore, met the definition of osteopenia, whereas seven (41.2%) had a T-score below −1 in LS. No significant difference was observed in T-scores or Z-scores of patients with mean Phe below 600 µmol/L and those exceeding the 600 µmol/L in the 5 years prior to study participation in hips (good compliance vs. poor compliance: T-score: −0.53 ± 1.22 vs. −0.79 ± 0.93, p = 0.613; Z-score: −0.39 ± 1.21 vs. −0.45 ± 1.12, p = 0.918, **Supplementary Table S4**) and LS (good compliance vs. poor compliance: T-score: −0.56 ± 1.18 vs. −0.84 ± 1.10, p = 0.618; Z-score: −0.40 ± 1.21 vs. −0.61 ± 1.17, p = 0.714, **Supplementary Table S4**).

### 3.4 Diet compliance and dietary behavior of patients with PKU in association with their bone health

Information on AA supplementation was missing from four patients with PKU, 13 patients with PKU took vitamin- and mineral-enriched AA supplements, whereas one patient did not consume AA supplements. Out of the 13 patients consuming AA supplements, 46.1% (n = 6) took supplements with glycomacropeptides (GMPs) (data not shown). Additionally, one patient was treated with BH_4_ and AA supplementation, and no patient was treated with Pegvaliase. The patients taking GMP were significantly older than those taking AA [GMP: 45.1 (42.5–48.0) years vs. AA: 23.8 (19.7–25.2) years; p = 0.002; r = −0.79].

During the last 5 years prior to study participation, three patients with PKU did not evaluate their blood Phe concentration. Out of the remaining 15 patients with PKU who had measured their blood Phe concentration, eight patients had mean blood Phe concentration below 600 µmol/L during the 5 years prior to study participation and were, therefore, classified as well compliant to treatment. Seven patients had mean blood Phe above 600 µmol/L during the 5 years prior to study participation and were, therefore, poorly compliant; as guidelines suggest, blood Phe concentrations in adults should stay below 600 µmol/L (1). The three participants who during the prior 5 years never evaluated their blood Phe concentrations were also grouped as poorly compliant.

In addition, to dietary compliance the patients with PKU also differed in some lifestyle characteristics. Although patients with good compliance had a significantly higher SES (good compliance: 8.3 ± 1.2 vs. poor compliance: 5.4 ± 2.1, p = 0.003, Hedges’ g = 1.57), they also smoked significantly less [pack-years: good compliance: 0 years (0–0) vs. poor compliance: 1.5 (0–4.5), p = 0.034, r = −0.55; **Table 3**; **Supplementary Table S3**] and walked further than expected during the 6MWT [good compliance: 55.9 m (10.1–173.3) vs. poor compliance −9.9 m (−139.4–37.4), p = 0.027, r = −0.52]. No difference was observed in age, BMI, or gender distribution, as well as WISH score (**Table 3**).

**Table 3:** Comparison of characteristics of the participating patients with Phenylketonuria with bone mineral density (BMD) T-score above −1 and below −1 in either hips or lumbar spine (LS).

Parameter	Hip BMD T-score		p	Effect size	LS BMD T-score		p	Effect size
	Over −1	Below −1			Over −1	Below −1		
n	9	8	–	–	10	7	–	–
**WISH**	**68.9 ± 11.8**	**49.5 ± 14.0**	**0.010**	**1.42^†^**	59.3 ± 19.4	59.1 ± 11.8	0.984	0.01^†^
Protein-rich foods (%#)	7 ± 5	17 ± 13	0.063	−0.95^†^	11 ± 11	13 ± 12	0.766	−0.14^†^
Phe during last 5 years (µmol/L)	543 ± 233	674 ± 531	0.526	−0.34^†^	672 ± 363	386 ± 255	0.180	0.79^†^
Ratio of all Phe measurements over 600 µmol/L	80 (10–100)	100 (0–100)	0.837	−0.07^⊖^	90 (15–100)	67 (0–100)	0.875	−0.04^⊖^

^#^percentage from sum of carbohydrate-rich and protein-rich foods; ^†^Hedges’ g; ^⊖^Pearsons r; 6MWT, 6-min walk test; ΔDistance 6MWT, difference between walked distance vs. predicted distance 6MWT (m); BMI, body mass index; SES, socioeconomic status; Phe, Phenylalanine; WISH, World Index for Sustainability and Health. Data are reported as median (25th–75th percentile) (non-normal distribution) or as mean ± standard deviation (normal distribution). Significant results are marked bold.

Patients with PKU and low hip BMD had a significantly lower WISH (p = 0.010; **Table 3**) than patients with BMD above −1. Here, patients with PKU and low hip BMD reached significantly less points in the unhealthy food section of WISH than patients with BMD above −1 (below −1: 7.5 ± 6.6 vs. above −1: 18.3 ± 6.4; p = 0.005; Hedges’ g = 1.58) and tended to reach lower points in healthy food section of WISH (below −1: 42.1 ± 9.2 vs. above −1: 50.6 ± 7.7; p = 0.065; Hedges’ g = 0.95). Additionally, patients with BMD below −1 tended to consume higher proportion of protein-rich foods (p = 0.063) than patients with BMD above −1 (**Table 3**), whereas none of these differences were observed in LS patients with high vs. low LS BMD. Patients with a LS BMD above −1 were significantly less often active during their everyday life, as suggested by results of the 6MWT. None of the other basic parameters were different between patients with PKU with high versus low LS BMD (**Table 3**; **Supplementary Table S4**).

### 3.5 Association of physiological, lifestyle, and disease parameters with bone mineral density in patients with PKU

In the regression analysis, only body weight was positively associated with hip and LS BMD, whereas neither lifestyle factors nor mean Phe during the last 5 years were associated with BMD (**Table 4**).

**Table 4:** Regression analysis on influencing factors bone mineral density (BMD) in hip and lumbar spine (LS).

Dependent variable	Block	Variables	B	Standard error^a^	95% CI		p
					Lower limit	Upper limit	
Hip BMD T-score	Physiological	Age (years)	−0.035	0.029	−0.097	0.026	0.238
		Height (cm)	−0.056	0.044	−0.151	0.039	0.225
		**Weight (kg)**	**0.049**	**0.009**	**0.029**	**0.069**	**<0.001**
	Lifestyle	WISH	0.046	0.025	−0.009	0.101	0.092
		SES	−0.071	0.128	−0.352	0.210	0.590
		Pack-years	0.090	0.163	−0.268	0.447	0.593
		ΔDistance 6MWT	0.006	0.005	−0.005	0.017	0.274
	Disease	Mean Phenylalanine last 5 years (µmol/L)	0.000	0.001	−0.003	0.002	0.748
Hip BMD Z-score	Physiological	Age (years)	−0.029	0.027	−0.087	0.029	0.301
		Height (cm)	−0.068	0.038	−0.149	0.012	0.090
		**Weight (kg)**	**0.054**	**0.010**	**0.033**	**0.075**	**<0.001**
	Lifestyle	WISH	0.050	0.026	−0.006	0.107	0.077
		SES	−0.155	0.158	−0.498	0.188	0.345
		Pack-years	0.074	0.188	−0.335	0.483	0.701
		ΔDistance 6MWT	0.004	0.005	−0.006	0.014	0.393
	Disease	Mean Phenylalanine last 5 years (µmol/L)	0.000	0.002	−0.004	0.003	0.862
LS BMD T-score	Physiological	Age (years)	−0.023	0.036	−0.101	0.056	0.546
		Height (cm)	−0.047	0.049	−0.153	0.059	0.353
		**Weight (kg)**	**0.036**	**0.013**	**0.007**	**0.064**	**0.019**
	Lifestyle	WISH	0.014	0.046	−0.088	0.115	0.774
		SES	0.045	0.219	−0.436	0.526	0.842
		Pack-years	0.017	0.183	−0.386	0.420	0.929
		ΔDistance 6MWT	00.000	0.007	−0.015	0.015	0.999
	Disease	Mean Phenylalanine last 5 years (µmol/L)	0.000	0.001	−0.001	0.002	0.550
LS BMD Z-score	Physiological	Age	−0.005	0.031	−0.071	0.062	0.878
		Height (cm)	−0.042	0.038	−0.124	0.040	0.293
		**Weight (kg)**	**0.037**	**0.013**	**0.009**	**0.065**	**0.012**
	Lifestyle	WISH	0.017	0.047	−0.085	0.118	0.729
		SES	0.060	0.194	−0.362	0.482	0.761
		Pack-years	0.043	0.220	−0.435	0.522	0.847
		ΔDistance 6MWT	0.001	0.007	−0.014	0.016	0.929
	Disease	Mean Phenylalanine last 5 years (µmol/L)	0.001	0.001	−0.001	0.003	0.550

ΔDistance 6MWT, difference between walked distance vs. predicted distance 6MWT (m); 6MWT, 6-min walk test. Significant results are marked bold. ^a^Corrected by the HC3 method.

## 4 Discussion

As the main symptom of PKU, neurologic damages, is minimized by treatment and patients are living a longer and healthier life, comorbidities have started to become prevalent. One of these comorbidities frequently described is a reduced BMD. About 40%–60% of patients with PKU are affected by low bone health, already showing first signs in childhood and adolescence (34, 35). The aim of our study was to determine the influence of nutrition, midterm dietary compliance, and lifestyle factors on bone health in patients with PKU. Whereas 47.1% of the participating patients with PKU met the criteria for osteopenia based on DXA assessment at the left hip, no differences in BMRP in patients with osteopenia and no-osteopenia or controls were detectable. A recent meta-analysis found similar results with BMD in patients with PKU being significantly lower than in the general population and 42% of patients being classified as low BMD (Z-score ≤ −1) (36). As previously described (37, 38), patients with PKU tend toward better 25-OH vitamin D status than the general population, which was also observed in this cohort. Patients with PKU and controls were included on the same day; therefore, seasonal differences in vitamin D status are unlikely to have led to higher 25-OH D concentration in patients with PKU. A higher vitamin D ingestion from AA supplements is probably the cause for the higher 25-OH D serum concentration (39). Nagasaka et al. reported higher bone resorption in patients with PKU and lower 25-OH D than controls (40). Compared to that study, patients in this study were older and had higher 25-OH D, which may explain why no differences in bone resorption were observed. However, BMD-results were comparable to the study by Nagasaka et al. (40). Whereas total body BMD was significantly reduced in Greek children and adolescents with PKU and also in LS BMD of children and adolescents with mild hyperphenylalaninemia, serum calcium (Ca), protein, and ALP were not different in these patients (8). Modan-Moses et al. observed BMD Z-scores in adult patients slightly lower than in these patients (34). In 183 patients with PKU from eight different metabolic centers in Europe, significantly reduced BMD Z-scores in LS, femoral neck, and total proximal femur were observed (9). These results were included into a recent meta-analysis that found significantly reduced LS BMD Z-scores in the pooled 304 adolescent and adult patients with PKU out of the 10 included studies (36). Patients in our study had comparably lower BMD Z-scores in LS and hip to the patients in the meta-analysis, although results missed significance, despite relatively normal BMRPs, probably due to the small sample size and the large range of measurements. Whereas skeletal changes that affect BMD take some time to become obvious, BMRPs represent actual changes in the activity and regulation of osteoblasts and osteoclasts *in situ* (41). A direct correlation of BMRPs and BMD is not guaranteed (41). Whereas some studies found relationships of some BMRPs and BMD in parts of the participants, others did not find any relationship of BMD and BMRPs (41–43).

However, even subclinical changes in BMRP can add up to changes in BMD over time (44–46). Therefore, lower BMD readings in the study participants could be a result of accumulated shortcomings in bone mass formation over several years before study participation. Several studies found reduced BMD or bone quality in pediatric and adolescent patients (35, 47–49), with low serum concentrations of bone-specific ALP, OCN, and Ca and lower urinary creatinine than controls and reduced bone turnover (45, 48). Tanaka et al., however, reported improvements in bone quality in participating children and adolescents with PKU, following only 34 days of Ca supplementation (47). The recommended initial treatment for low BMD in adolescents with PKU consists out of optimization of diet and physical activity, followed by further analysis of other factors and treatments in case of persistent low BMD measures (1). The exact onset of impaired skeletal mineralization in patients with PKU is unclear and further research is needed to evaluate the onset of bone mass reduction in patients with PKU to start treatment as early as possible.

As in the general population (50), there was a trend toward better BMD with higher BMI in the present study, whereas Doulgeraki et al. did not find differences in BMI between patients with PKU and controls or correlation between BMI and BMD (8). As BMD accrual depends on muscle-bone interaction, reduced muscle mass in favor of higher fat mass could have led to reduced bone mass accrual in the patients with PKU. Choukair et al. observed significantly reduced volumetric total BMD in the radius in a group of 56 adolescent and adult patients with PKU. This finding was associated with reduced grip strength, but the association was weaker than in controls. Therefore, the authors concluded that, in addition to a reduced muscle size, the adaptation of bones to loading is reduced in patients with PKU (51). In the present study, patients with PKU had similar everyday activity compared to controls, but a weaker performance in the 6MWT. Others observed a significantly reduced BMD and maximal capacity in incremental workload in patients with PKU who discontinued AA supplements compared to healthy controls, whereas patients who adhered to the dietary treatment only showed a slightly reduced maximal capacity (11). Furthermore, lower lean body mass Z-scores than expected were observed in adolescents and young adults with PKU who had blood Phe over 600 µmol/L (52). Reduced muscle accrual during adolescents and early adulthood could have led to reduced bone mass accrual in the patients with PKU, but we are lacking data on lean body mass and 6MWT might not have been sensitive enough to detect differences in this cohort. As in adolescents and young adults with high blood Phe concentrations, lean body mass of adolescents with inherited AA metabolism disorders other than PKU also was significantly smaller than in controls (53). In a small study population (n = 15), men with PKU had low-normal lean body mass, whereas women with PKU had significantly higher body fat mass than men (54). In a larger study population, patients with non-classical PKU (n = 38) not only showed a significantly higher proportion of body fat than patients with classical PKU (n = 58) but also were taller than these, which was predicted by Phe concentration in serum (14). Reduced protein consumption could lead to the observed changes in body composition. Therefore, Evans et al. identified a sweet spot of 12%–18% of energy from all protein sources combined to ensure healthy growth and reduced body fat mass of children with PKU (55). As the diet of patients with PKU excludes most animal foods such as meat, dairy products, and fish due to their high protein content, one could argue that the diet is comparable to that of vegans. A meta-analysis of studies with vegan children reported lower bone mineral content (BMC), smaller height, and lower protein intake, although recommended ranges for body height, calcium, and protein intake were still attained (56). The amount of protein consumption is discussed to influence the development of osteoporosis and its progression. Current literature on the consumption of milk and dairy products suggests that a higher intake is beneficial, whereas the consumption of plant proteins seems not to be detrimental as long as an adequate amount of micronutrients such as Ca and vitamin D are consumed (57). Furthermore, Kędzia et al. discussed a benefit for BMD in case of carbohydrate reduction in favor of protein consumption especially in women (57). As patients with PKU need to reduce their intake of natural protein, they consumed a higher proportion of carbohydrate-rich foods in this study population. Inappropriate protein supply by reduced supplement intake in some patients could, therefore, account for some reduction in BMD, as previously reported in 43 patients with PKU (58), as well as in patients with inherited AA metabolism disorders other than PKU (53). As we were unable to calculate the exact uptake of protein from natural protein and supplements, we cannot conclude if reduced protein supply at some point during the patient’s life has negatively affected BMD, even though the amount of natural protein rich foods consumed by the patient was lower than in controls.

Overall, we did not observe differences in nutrition between controls and adult patients with PKU in WISH, but major food groups like milk and meats were frequently substituted by patients with PKU, which did not influence WISH. We observed higher WISH in patients without osteopenia than in patients with osteopenia. As WISH can be split into an unhealthy subsection and a healthy subsection (24), we applied these subsections to analyze differences in nutrition of patients with and without osteopenia. Patients with osteopenia consumed higher amounts of foods in the unhealthy subsection of WISH (red meat, saturated fats, sugar sweetened beverages, sugary and salty snacks, and spreads) than patients with higher BMD. A negative association of BMD or BMC and unhealthy food patterns has been described previously (59). As high salt intake could negatively impact calcium metabolism, salty snacks are advised to be consumed in low amounts (19). In a large cohort of older adults (n = 4,028), adherence to a food pattern with higher amounts of fruits, vegetables, and dairy products was associated with a lower risk of osteoporotic fractures, whereas adherence to a food pattern defined by sweets, animal fat, and low meat was associated with a higher hazard for these (60). Additionally, a 2021 meta-analysis found a significant inverse association between the intake of sugar-sweetened beverages and BMD, especially in adults younger than 30 years old (61).

Previously, it was suggested that elevated Phe concentrations could influence bone health negatively (10, 52, 62, 63). However, there was no correlation of mean blood Phe in the year prior to study participation and BMD in two studies on patients with PKU (34, 64). Similarly, we did not observe differences in mean Phe in the 5 years prior to participation in patients with PKU with osteopenia and those without osteopenia or significant result in the regression analysis. Coakley et al. found no significant association between dietary Phe:tyrosin ratio, but a significant influence of compliance to diet and BMD Z-score (49). As almost 70% of these study participants were underage, a stricter dietary regime and a broader deviation of dietary regimes can be expected than in participants of the present study. Furthermore, a significantly higher current Phe serum concentration was observed in patients with PKU than in patients with untreated hyperphenylalaninemia, without a difference in BMD between these groups (14). Roato et al. observed a reduced phalangeal quantitative ultrasound (a parameter for bone quality) in patients with Phe blood concentration over 600 µmol/L (10). However, these patients were also younger than the participants of this study, especially those with low Phe concentration (10).

Next to blood Phe influencing bone mass, medical foods might influence bone mass as well. Acid load on kidneys is discussed to have a negative effect on BMD in patients with PKU, and, previously, a very high acidity of low Phe preparations was a problem in treatment (16, 65). Up until today, higher renal acid load is observed in patients with PKU consuming AA supplements, compared to patients consuming GMP (16). After lifelong AA consumption, a switch to GMP resulted in lower urinary Ca and magnesium excretion (16). Additionally, male patients with PKU consumed higher amounts of AA and showed higher renal Ca excretion and simultaneously lower BMD Z-scores than female patients with PKU (54). Therefore, higher renal loss of Ca is discussed to result in reduced BMD in patients consuming AA mixtures (16, 66). Because only few patients, who were significantly older than the patients taking AA, were consuming GMP in this study, BMRPs were not compared between these groups.

### 4.1 Limitations

Despite intensive efforts to recruit, the number of participating patients was quite small, making comparisons between subgroups impossible. As loss to follow-up in adults with PKU is a large issue (21), not only in Germany (67), recruitment in additional specialized clinics would be needed for further analyses. Additionally, it was not possible to obtain DXA scans for healthy controls; thus, the results were solely compared to a reference population. We calculated a BPH to account for this problem, classifying participants by BMRP within and out of reference margins. Moreover, as this was a retrospective cross-sectional study, we cannot determine whether the reduced BMD observed in the patients with PKU persisted throughout their lives or developed only recently. Furthermore, Phe blood concentrations in the 5 years prior to study participation were only available for some of the study participants, probably because some participants were no longer in regular care of a specialized center. Regrettably, we do not have data on early childhood treatment, information on genetic mutation or time of diagnosis. Because we have data on Phe concentration or treatment of 17 patients with PKU, all of these can be classified as having classical PKU. Additionally, only usual foods could be included into the calculation of the WISH score (24, 25); therefore, a potential influence of different AA supplements or specialized food could not be considered in the evaluation of the usual diet. Because adult patients with PKU tend to eat a vegan-like diet with a wider variety of natural foods than children (1, 5), we did not observe a difference between healthy adults and patients with PKU in regards of the WISH score.

## 5 Conclusion and perspective

In summary, almost half of the participating adults with PKU had reduced BMD in the left hip on DXA examination, but no differences in BMRP or lifestyle factors were seen in patients with osteopenia, without osteopenia or in controls. In addition, dietary adherence in the 5 years prior to study participation was not associated with observed BMD. Further studies are needed to assess the first occurrence of bone loss in patients with PKU and to optimize treatment options. Further improvements in AA supplementation could lead to better outcomes, like it has been observed with some GMP products. In addition, other mediating factors such as the gut microbiome, low muscle mass, or low-grade inflammation could influence the formation and resorption of bone mass and require future research.

## Data Availability

The raw data supporting the conclusions of this article will be made available by the authors, without undue reservation.
