# Supplementary material for: Adults with Phenylketonuria have suboptimal bone mineral density apart from vitamin D and calcium sufficiency
Source: Front Endocrinol (Lausanne). 2025 Feb 14;16:1488215. doi: 10.3389/fendo.2025.1488215 (PMC11867911; doi:10.3389/fendo.2025.1488215)
Supplement: Supplementary file 1 [file Table1.docx]

Supplementary Material

**Adults with Phenylketonuria have suboptimal bone mineral density apart from Vitamin D and calcium sufficiency**

**Table S 1:** Comparison of height, weight and body mass index (BMI) of general German population [33], patients with phenylketonuria (PKU) and controls (Co).

|  |  | **PKU** | | | **Co** | | |
| --- | --- | --- | --- | --- | --- | --- | --- |
|  |  | **Mean** | **p^#^** | **Effect size** | **Mean** | **p^#^** | **Effect size** |
| Height (cm) | Female | 161.9 | 0.088 | -0.53 | 169.5 | 0.064 | 0.61 |
|  | Male | 175.7 | 0.184 | -0.42 | 184.0 | 0.072 | 0.62 |
| Weight (kg) | Female | 66.1 | 0.633 | -0.14 | 66.6 | 0.459 | -0.22 |
|  | **Male** | **76.4** | **0.007** | **-1.30** | 87.3 | 0.640 | 0.15 |
| BMI | Female | 25.1 | 0.958 | -0.02 | 23.2 | 0.156 | -0.45 |
|  | **Male** | **24.8** | **0.039** | **-0.86** | 25.9 | 0.488 | -0.29 |

^#^one sample t-test^, †^Hedges’ g. BMI body mass index. Significant results are marked bold.

**Table S 2:** Serum parameters of oxidative stress and bone metabolism in adult patients with phenylketonuria (PKU) and controls (Co).

|  | **PKU** | **Co** | **p** | **Effect size** |
| --- | --- | --- | --- | --- |
| Phosphate (mg/dL) | 1.19 ± 0.22 | 1.21 ± 0.15 | 0.755 | -0.10^#^ |
| Ca (mmoL/L) | 2.4 [2.4 – 2.5] | 2.5 [2.4 – 2.5] | 0.538 | -0.11^Ꝋ^ |
| CRP (mg/dL) | 0.130 [0.042 – 0.333] | 0.042 [0.042 – 0.150] | 0.126 | -0.26^Ꝋ^ |
| Lipidperoxides (µmoL/L) | 192.0 [76.5 – 395.5] | 74.0 [38.0 – 225.0] | 0.313 | -0.17^Ꝋ^ |
| Vit C (mg/L)* | 10.4 ± 3.5 | 11.8 ± 3.7 | 0.253 | -0.39^#^ |
| Vit E (µg/mL)* | 12.0 [11.15 – 14.95] | 12.6 [10.6 – 14.4] | 0.802 | -0.05^Ꝋ^ |
| Crea | 0.918 ± 0.141 | 0.871 ± 0.137 | 0.321 | 0.34^#^ |

*PKU: n = 17; Ca calcium; Crea creatinine; CRP c-reactive protein; Vit C Vitamin C; Vit E Vitamin E. Data are reported as median (25–75 percentile) (non-normal distribution) or as mean ± standard deviation (normal distribution). ^#^Cohen’s d; ^Ꝋ^ Pearsons r

**Table S 3:** Comparison of BMRP, BMD and oxidative stress parameters between patients with Phenylketonuria (PKU) and good compliance to diet (defined by mean blood Phe below 600 µmol/L during the 5 years prior to participation) and patients with PKU and poor compliance (defined by mean blood Phe above 600 µmol/L during the 5 years prior to participation or no measurements during the 5 years prior to participation)

| **Parameter** | **Phe during 5 years prior to study participation** | | **p** | **Effect size** |
| --- | --- | --- | --- | --- |
|  | Good compliance | Poor compliance |  |  |
| n | 8 | 10 | - | - |
| **Serum** | | | | |
| ALP (U/L) | 67.0 [58.8 – 79.8] | 81.0 [54.3 – 92.5] | 0.573 | -0.15^Ꝋ^ |
| IGF1 (ng/mL) | 225.4 ± 90.0 | 212.4 ± 71.0 | 0.744 | 0.153^†^ |
| OCN (ng/mL) | 19.1 [15.6 – 22.9] | 21.3 [19.9 – 25.9] | 0.122 | -0.38^Ꝋ^ |
| OPG (pg/mL) | 110.3 ± 25.9 | 132.4 ± 43.8 | 0.233 | -0.57^†^ |
| TRAP (U/L) | 2.20 ± 0.99 | 2.71 ± 0.71 | 0.237 | -0.57^†^ |
| 25-OH D (ng/mL) | 33.8 ± 7.7 | 33.3 ± 11.9 | 0.919 | 0.05^†^ |
| Phosphat (mg/dL) | 1.19 [0.94 – 1.24] | 1.21 [1.03 – 1.42] | 0.515 | 0.17^Ꝋ^ |
| Ca (mmol/L) | 2.40 [2.33 – 2.50] | 2.45 [2.40 – 2.50] | 0.633 | -0.12^Ꝋ^ |
| CRP (mg/dL) | 0.06 [0.04 – 0.17] | 0.21 [0.09 – 0.45] | 0.083 | -0.41^Ꝋ^ |
| Lipidperoxides (µmoL/L) | 153.0 [34.5 – 278.5] | 238.0 [75.3 – 937.1] | 0.274 | -0.27^Ꝋ^ |
| **Vit C (mg/L)** | **12.3 ± 2.7** | **8.7 ± 3.4** | **0.031** | **1.10**^†^ |
| Vit E (µg/mL) | 12.6 ± 3.5 | 12.9 ± 2.4 | 0.858 | -0.08^†^ |
| **Plasma** | | | | |
| CTX (pg/mL) | 272.1 ± 183.1 | 321.3 ± 169.2 | 0.577 | -0.27^†^ |
| PTH (pg/mL) | 25.8 [19.6 – 45.2] | 28.5 [19.4 – 42.4] | 0.962 | -0.02^Ꝋ^ |
| **Urine** | | | | |
| Pyr/Crea (µg/g Crea) | 135.6 ± 50.9 | 181.5 ± 119.4 | 0.330 | -0.47^†^ |
| DPD/Crea (µg/g Crea) | 26.3 [19.0 – 32.7] | 25.8 [19.5 – 54.0] | 0.815 | -0.06^Ꝋ^ |
| Phosphat (mmol/L) | 6.85 [3.75 – 13.00] | 10.90 [6.80 – 22.05] | 0.139 | 0.03^Ꝋ^ |
| Ca/Crea (mmol/g Crea) | 5.61 ± 3.54 | 5.69 ± 4.36 | 0.969 | -0.02^†^ |
| BPH | 0.00 [0.00 - 0.17] | 0.17 [0.00 – 0.38] | 0.237 | 0.04^Ꝋ^ |
| **BMD** | | | | |
| Hip T-Score | -0.53 ± 1.22 | -0.79 ± 0.93 | 0.613 | 0.24^†^ |
| Hip Z-Score | -0.39 ± 1.21 | -0.45 ± 1.12 | 0.918 | 0.05^†^ |
| LS T-Score | -0.56 ± 1.18 | -0.84 ± 1.10 | 0.618 | 0.24^†^ |
| LS Z-Score | -0.40 ± 1.21 | -0.61 ± 1.17 | 0.714 | 0.17^†^ |

^†^Hedges’ g; ^Ꝋ^Pearsons r; 25-OH D 25-hydroxy Vitamin D; ALP total alkaline phosphatase; BPH Bone pathology harbinger; Ca calcium; Crea creatinine; CRP c-reactive proteine; CTX cross-linked C-telopeptide of type I collagen; DPD deoxypyridinoline; IGF1 insulin-like growth factor; LS lumbar spine; OCN osteocalcin; OPG osteoprotegerin; PTH intact parathyroid hormone; Pyr pyridinoline; TRAP tartrate-resistant acid phosphatase 5b; Vit C Vitamin C; Vit E Vitamin E. Data are reported as median (25–75 percentile) (non-normal distribution) or as mean ± standard deviation (normal distribution). Significant results are marked bold.

**Table S 4:** Characteristics of comparison of characteristics of the participating patients with Phenylketonuria with bone mineral density (BMD) T-Score above -1 and below -1 in either hips or lumbar spine (LS).

| Parameter | Hip BMD T-Score | | p | Effect size | LS BMD T-Score | | p | Effect size |
| --- | --- | --- | --- | --- | --- | --- | --- | --- |
|  | over -1 | below -1 |  |  | over -1 | below -1 |  |  |
| n | 9 | 8 | - | - | 10 | 7 | - | - |
| Age (years) | 34.5 ± 12.5 | 37.9 ± 10.2 | 0.548 | -0.28^†^ | 39.5 [24.2 – 48.0] | 38.6 [23.8 – 44.3] | 0.601 | -0.14^Ꝋ^ |
| Female n (%) | 5 (56%) | 5 (63%) | 1.000 | - | 7 (70%) | 3 (43%) | 0.350* | - |
| Height (cm) | 167.1 ± 10.2 | 169.5 ± 6.9 | 0.585 | -0.26^†^ | 167.6 ± 9.2 | 169.1 ± 8.4 | 0.729 | -0.17^†^ |
| Weight (kg) | 76.3 ± 20.9 | 64.3 ± 11.1 | 0.166 | 0.67^†^ | 74.1 ± 20.7 | 65.7 ± 11.9 | 0.351 | 0.45^†^ |
| BMI (kg/m²) | 27.2 ± 6.8 | 22.2 ± 2.3 | 0.067 | 0.91^†^ | 23.9 [21.8 – 29.0] | 22.3 [21.7 – 24.7] | 0.270 | -0.28^Ꝋ^ |
| Pack-years | 0.0 [0.0 – 2.5] | 0.5 [0.0 – 3.5] | 0.517 | -0.16^Ꝋ^ | 0.0 [0.0 – 4.0] | 0.0 [0.0 – 2.0] | 0.956 | -0.01^Ꝋ^ |
| Not fit 6MWT n (%) | 4 (44%) | 1 (13%) | 0.294* | **-** | **5 (50%)** | **0 (0%)** | **0.044*** | **-** |
| ΔDistance 6MWT | 22.8  [-62.3 – 127.3] | 23.6 [5.5 – 62.6] | 1.000 | -0.02^Ꝋ^ | -1.1 [-95.8 – 97.0] | 35.7 [5.8 – 74.6] | 0.417 | -0.21^Ꝋ^ |
| SES | 7.2 ± 1.7 | 6.7 ± 2.2 | 0.575 | 0.26^†^ | 7.0 ± 1.7 | 6.9 ± 2.3 | 0.961 | 0.02^†^ |
| Smoking habits n (%) |  | | | | | | | |
| Never | 6 (67%) | 4 (50%) | 0.876* | - | 6 (60%) | 4 (57%) | 0.347* | - |
| Not any more | 1 (11%) | 1 (13%) |  |  | 0 (0%) | 2 (29%) |  |  |
| Sometimes | 1 (11%) | 2 (25%) |  |  | 2 (20%) | 1 (14%) |  |  |
| Daily | 1 (11%) | 1 (13%) |  |  | 2 (20%) | 0 (0%) |  |  |
| Heavy smoking according to RKI [26] | 1 (11%) | 1 (13%) | 0.718* | - | 2 (20%) | 0 (0%) | 0.485* | - |
| Females that were pregnant before n (%) | 1 (20%) | 2 (40%) | 1.000* | - | 2 (40%) | 1 (33%) | 1.000 | - |

^†^Hedges’ g; ^Ꝋ^Pearsons r; *fishers exact test. 6MWT 6-minute walk test; ΔDistance 6MWT Difference between walked distance and predicted distance 6MWT (m); BMI body-mass index; RKI Robert Koch-Institute; SES socio economic status; WISH World Index for Sustainability and Health; Phe Phenylalanine. Data are reported as median (25–75 percentile) (non-normal distribution) or as mean ± standard deviation (normal distribution). Significant results are marked bold.
